# Identifying a Framework for Hope in Order to Establish the Importance of Generalised Hopes for Individuals Who Have Suffered a Stroke

**DOI:** 10.1155/2014/471874

**Published:** 2014-06-29

**Authors:** Andy Soundy, Clive Liles, Brendon Stubbs, Carolyn Roskell

**Affiliations:** ^1^School of Sport, Exercise and Rehabilitation Sciences, University of Birmingham, Birmingham B15 2TT, UK; ^2^School of Health and Social Care, University of Greenwich, London SE10 9LS, UK

## Abstract

Hope and hopelessness are important psychological constructs that physiotherapists should consider when working with patients who have experienced a stroke. The view of hope in rehabilitation is often focused around the concept of goals and how hope works within this framework. However, the current paper proposes a broader framework for hope and the importance of a more generalised view of understanding why a certain hope exists or is identified by a patient. A narrative review using an a priori thematic analysis was undertaken to consider how more generalised hopes are expressed by individuals who have suffered a stroke. An electronic search of 4 databases from inception until April 2014 was undertaken. Qualitative articles were included if they considered the concept of hope for patients who had suffered a stroke. The results identified three themes which included (1) consideration of the patient's identity/identities, (2) meaningful activities, experiences, and interactions, and (3) the experience of suffering and need for relief. An awareness of patients' generalised hopes should be a priority for HCPs. Detailed implications for HCPs are identified within the discussion.

## 1. Introduction

The most accepted definition of hope within positive psychology [1] is generated from Snyder et al. [2] which states that hope is “*a positive motivational state that is based on an interactively derived sense of successful (a) agency (goal directed energy), and (b) pathways (planning to meet these goals)*” (page 287). Hope can be seen as an essential part of recovery for patients with a chronic illness [3] and is a very important concept for individuals who have suffered a stroke [4, 5]. However, there are several factors which challenge the hope of an individual following their stroke [6]. For instance, hope is severely challenged at times of disease onset, during change [7], or uncertainty [8]. It is particularly important if progress through rehabilitation is slow [9] or if individuals do not achieve what they had expected [10] and it is also severely challenged by individuals feeling dependent on others [11]. Importantly, if hope is lost, it can render patients vulnerable to severe consequences such as major depression [4] and, as reported in other neurological conditions, can end in suicide [12]. Whilst there has been an increase in research considering hope in individuals with a stroke, there is a lack of clarity in how the concept is understood in this population [5].

**Figure 1:**
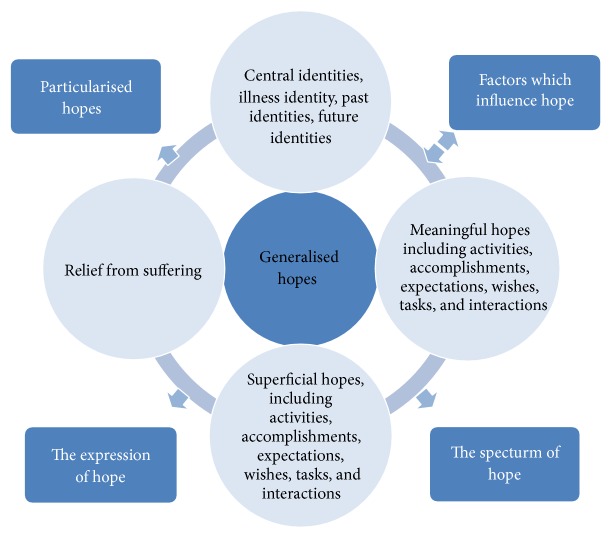
Illustrating the framework of hope.

Goals are used by health care professionals as a key way of managing which is important to patients [13]. Thus, Snyder's definition of hope has great value in rehabilitation. However, it may be that, in a pressured environment, with limited time between patients, functional improvements [14] and institutional goals [15] (politically orientated “tick box” style goals) are the main or sole goals that are documented. However, if health care professionals can think past this view of goals and achievement, rehabilitation has the potential to provide a much deeper influence on an individual's life. For instance, understanding a patient's social identity/identities is extremely important, since multiple group membership, especially if it is established before stroke, is associated with greater life satisfaction and well-being after stroke [16]. Taking other factors that contribute to hope into consideration, a broader framework needs to be considered that conceptualises hope for chronic illness. We establish a framework for hope below and identify how current stroke literature on hope fits this framework. A summary of the conceptualisation of hope is provided in Figure 1 and each element is identified below.

### 1.1. Generalised Hopes, Orders, and Levels of Hope

Wiles et al. [3] split hope into particularised hope and generalised hope. Particularised hopes have received far more attention in the research literature, including stroke [13], as within research they are often those which focus on goals and recovery or outcomes that are possible following treatment [17]. It is important because, in order for a patient to keep persisting with rehabilitation, they need to be able to see a valued future often expressed in the form of a goal [18]. Snyder's hope theory [2, 17, 19] focuses on the importance of particularised hope and goals and dominates the rehabilitation landscape, by requiring health care professionals to manage hope by working with goals, with review evidence supporting the value of goals for stroke patients [13].

By contrast, the hope concept is used differently by researchers working in chronic illness [20–22]. Indeed, it has been suggested that hope is more than focusing individuals on a particular goal. For instance, Barnard [21] highlights, when commenting on Marcel's view of hope, “*hoping is a posture, not a motive for achievement of a particular goal. It is a mode of experiencing oneself in relation to reality and time*” (page 47). Further, it is important to acknowledge that individuals' hopes may vary. Researchers split generalised hopes into levels or intensities [23] or orders [20]. At the most intense level, future hopes and expectations may be prevented by the experience of suffering. During experiences of suffering individuals may only be able to hope for the suffering to stop [23]. Once this has happened, an opportunity may be presented for the individual to turn towards a hope that is not yet perceived [20].

Part of what gives life meaning for individuals is their identity/identities; for instance, Marcel [20] states that* there are orders of hope from low superficial hopes to higher orders of hope. *In relation to superficial hopes he states, “*[the] reasons for hoping are external to the self…far from having their roots in the very depths of what I am*” (page 29). This signifies that with higher order hopes (hope that is integral to one's identity) the reason for hope is at the very depths of who the patient is. This links in with the definition of generalised hopes [3] as “*a state that gives life meaning and protects against despair*” (page 565). Linked with this, at a lower level or order of hope are the meaningful activities, work, or tasks that individuals undertake [6]. More explicit examples of the lower order or levels of hope are considered by Miller [23] which include two levels, in her terms, hopes around self-improvement, accomplishments, and relationships, as well as superficial wishes.

Importantly, the current paper is built on the premise that health care professionals would benefit from understanding these different orders and importantly from higher levels of hope, including a patient's identity and activities which have meaning to them, if they are to understand the goals generated within rehabilitation. That is, the identities a patient had before the illness provided meaning for their life and it may be that the goal of particularised hopes during rehabilitation is to restore these identities. Thus, identity is infused into a patient's hope and indeed linked to meaningful activities that the patient could reengage in. The role and importance of identity for patients can be observed in previous research considering chronic illness [24–26], as well as research considering individuals who have had a stroke. For instance, individuals who have suffered a stroke have a worse sense of self compared to their prestroke self [27].

### 1.2. Factors Which Influence Hope

Recent review evidence has identified different factors which influence the hope of individuals who have suffered a stroke [6] which found that hope is dependent on many internal, external, situational, interactional, and environmental factors [6]. For instance, internal factors may include not having goals in place or feeling captive to the illness and not being able to see a way out [20, 23]. Alternatively, interactional factors may play an important role; for instance, health care professionals may focus on or be concerned with obtaining and promoting a “realistic” hope for patients [28]; in contrast, patients can focus on obtaining hope through possibility that exists [4]. These different perspectives can lead to a negative interaction having the potential to remove a patient's hope, which in turn can have a lasting and significant impact on their health and well-being [29].

### 1.3. The Expression of Hope and Spectrum of Hope

In order to understand the expression of hope it is important to consider the work of Barnard [21]. Barnard [21] states that human existence gives birth to hope as we are poised on a boundary between finitude and transcendence and he uses the paradox of chronic illness to describe this:

*people with chronic conditions are impelled at once to defy limitations in order to realise greater life possibilities and to accept limitations in order to avoid enervating struggles with immutable constraints. This is the dialectical nature of chronic illness* (1995, page 39).

Viewing a patient's response to illness as expressed as a paradox demonstrates several important considerations for the terms acceptance and defiance [4, 21, 29]. What a health care professional may hear from a patient could be recognised by a certain narrative (a story that relates to the health and illness experiences of individuals), for example, the need to be restored to their former health, commonly labelled the restitution narrative [4]; this narrative illustrates an inability to accept what has happened and a defiance and a response which illustrates a concrete hope. Where health care professionals are able to understand the expression of hope, often in the form of illness narratives, they are able to gain information about a patient's hope and adjustment implicitly [4].

Finally, a spectrum of hope has been suggested within neurological literature [4] that identifies concrete hope representing one end of the spectrum of hope and at the other no hope or hopelessness. A concrete hope [21] is often expressed as a concrete wish “*I hope that I recover…these “hopes” are more properly seen as ardent wishes*” (page 47). Concrete hope is often combined with an idea of restoration of one's former self [4] and can be heard or expressed as an uncompromising position, which can be problematic [3, 30]. No hope or hopelessness is defined by key characteristics and feelings which include a sense of impossibility, the inability to see a way forward or way out [31]. It causes an individual to feel that nothing can be gained from a situation and thus there is no point in expending any energy in an attempt to change one's situation [32].

If a spectrum of hope was drawn up, the term possibility would represent a mid-point of the spectrum and be moderated by uncertainty of what is hoped for. Hope in possibility [4] represents a patient who has been able to reconcile their present circumstances to what the future may have been. Simultaneously, this acknowledges and allows the patient's defiant attitude, affording it a unique position in terms of the paradox of chronic illness, as well as representing a milestone of achievement in terms of how adjustment is expressed; for example, it represents the final stages in phase and stage models of adjustment [33]. This expression of hope and the spectrum of hope have been researched previously in stroke [4].

Given the above information it is clear that within stroke literature the proposed framework is supported by previous research studies, although further work is required to review literature that considers if stroke research on hope identified generalised hopes. Thus the purpose of the current review is to complete a framework on hope, focused on individuals with a stroke, by examining evidence for generalised hopes within previous literature.

## 2. Methods

### 2.1. Information Sources and Search Strategy

Four electronic databases were searched from inception until April 2014: AMED, CINAHL Plus, Medline (revised), and EMBASE. Hand-searching of the included paper's reference lists was employed and where possible authors were contacted when a study could not be located. The key words used included hope and review or illness or expectation or experience or recovery, and stroke.

### 2.2. Eligibility Criteria for the Review

The SPIDER tool (sample, phenomenon of interest, design, evaluation, and research type) was used to identify eligible studies. (1) In sample, individuals had to have suffered a stroke. (2) In phenomenon of interest, articles had to include a focus on the concept of hope or significantly discuss it in their results section. (3) In design, only qualitative articles were considered. (4) In evaluation, articles had to consider the attitudes, views, or experiences of patients. (5) In research type, only qualitative articles were included.

### 2.3. Critical Appraisal

The primary author utilised the consolidated criteria for reporting qualitative studies (COREQ) [34] to assess the quality of the included studies. The COREQ has three domains (research team and reflexivity, study design, and analysis and findings), as well as a total score. The score is based on each question either being reported correctly (scoring a point) or not (scoring no point), with a maximum possible score of 32.

### 2.4. Analysis

A thematic analysis of qualitative research [35] using a broad a priori structure of examining generalised hopes (identified in Figure 1) was conducted in three stages set out below.

## 3. Results

### 3.1. Search Outcome

A total of 10 studies met the inclusion criteria [7, 8, 10, 11, 36–41]. This included 112 people who had experienced a stroke (44 male, 68 female). The mean age of participants was ranged between 50 and 79.8 years.  Table 1 summarises the key characteristics of each study.

### 3.2. Critical Appraisal

The COREQ scoring system did not identify any studies as fatally flawed and in that the data generated within the results section could not be used for the purposes of this review.  Table 2 provides a summary score for each article. Full details of this can be obtainable from the primary author.

### 3.3. Thematic Analysis

Three themes were directly related to the “generalised hopes” section of our proposed framework. These included (1) consideration of the patient's identity/identities, (2) meaningful activities, experiences, and interactions, and (3) the experience of suffering and need for relief. A supplementary table in the Supplementary Material available online at http://dx.doi.org/10.1155/2014/471874 provides full details of the thematic breakdown. This includes details of the sub-themes and units (data identified from the the results sections of the included studies).

#### 3.3.1. Consideration of the Patient's Identity/Identities

Five subthemes were identified within this theme. First, individuals identified a loss of identity. This was highlighted by the general impact and loss across biopsychosocial domains and in different environmental settings. For some participants the stroke had removed who they were before stroke and this challenged specific, important identities and roles, for example, a role as an income provider, as a husband or wife, and as a mother. The study by Kouwenhoven et al. [40] highlights this, as one participant stated, “*Ingrid felt that she was not an efficient housewife anymore.… now her abilities were limited … She described herself as an old lady*.” Importantly, their role and identity, following the functional and physical change and loss from the stroke, were often characterised by a dependence on others. Second, patients described in great detail instances and changes that occurred from the stroke that created a different and distinct current identity for them. This was highlighted by the variation and requirement of dependency for assistance by others, changes to individual conditions, concentration, and personality. The fourth and fifth subthemes were interrelated together and included restoring their past identity or obtaining an identity that was valued and returning to their “normal” lives. As these themes suggested it was important for individuals to reestablish important identities which defined who they were prior to the stroke; this included all types of different identities like a previous job role or a role like being a mother. These roles were firmly related to what individuals considered normal and their prestroke life. For instance as Arnaert et al. [8] summarise, “*All participants expressed a future goal or desire…which collectively was the desire to return to normal pre-stroke life. This recurrent idea was indicated though a statement about the importance of work, school and leisure activities*.” Although one study [10] stated that this was not always needed, “*Matthew…didn't want to be the person he was before thestroke and thus, at one level, welcomed the chance to do thingsdifferently*.” Finally, some studies identified the importance of a religious identity for individuals which could aid their hope. This hope was generated from the guidance and support attributed to God (from the Christian faith).

#### 3.3.2. Meaningful Activities, Experiences, and Interactions

This theme included five subthemes. First, most studies identified the importance of meaningful interactions and frequently the very positive effect they could have on an individual's health. This was summarised well by Cross and Schneider [41] who stated, “*Support systems include medical staff, rehabilitation therapists, family, and friends. They are an intricate network that work together to motivate, inspire, guide, and care for the stroke survivors*.” However, the effects of the stroke could reduce the network available for meaningful interactions and interactions could also be negatively influenced by the stroke. Second, individuals wanted to return to meaningful activities, which included basic functions like walking, hobbies, and work related tasks. This included any activity that may have been undertaken before stroke and often was highly linked to the individual prestroke identity/identities. Although, functionally this not possible for all stroke survivors. Third, some patients highlighted that they had to redefine what was meaningful to them and appreciate it. Here they reframed what was important to them, becoming more content as illustrated by Kouwenhoven et al. [40], “*They became aware of what they described as the important things in life: being grateful for the things they have achieved, taking care of own health, and being together with their loved ones*.” Fourth, for some individuals, meaning was found in relation to their faith and finally individuals could highlight the meaning and hope associated with activities within their daily routine including the rehabilitation they received.

#### 3.3.3. The Experience of Suffering and Need for Relief

This theme included two subthemes. First, the experiences of suffering were identified as creating shock at the reality of what the stroke meant for individuals' lives. Individuals were described as being completely overwhelmed, overcome by different emotions and reactions to the stroke. For instance, this was summarised by Hartigan et al. [7] stating that “*Initially they [patients] expressed feelings of shock and fear related to loss of bodily control*.” Second, patients identified the impact of the stroke on what their future identities or roles would be; for some this meant having to wait and try out what the future may be like once home from rehabilitation; for others level of uncertainty about their prognosis prevented this. Still for others, the reality of living with impairments generated from the stroke was challenging and made the prospect of being discharged at times very alarming.

## 4. Discussion

The current results provide general support for the proposed framework which illustrates a broader consideration of the concept of hope. The findings of the current paper focused on the generalised hopes expressed by patients who have suffered a stroke. These hopes were significantly related to an individual's identity, as well as meaningful activities and interactions. This highlights the importance of the previous identity/identities and activities as a prime source of hope for patients with a stroke. It is important to consider that rehabilitation paves the way for individuals to begin considering how they can continue living after diagnosis/illness onset or symptom change. Indeed, rehabilitation provides grounding for allowing patients an understanding of how they can retain their independence, identity, and integrity as an individual. These findings are particularly important, since previous literature has highlighted the importance of meaningful identities, activities, and interactions on the adjustment of individuals who have suffered a stroke [6, 42], even years after the stroke event [43].

### 4.1. Acceptance and Generalised Hopes around the Patient's Identity/Identities or Meaningful Activities

Health care professionals need to understand how acknowledgement and acceptance are considered and expressed by patients. For instance, Marcel [20] highlights the importance of lived experience as a factor that contributes to acceptance and Charmaz [44] states that acceptance changes with experience, prospects, and plans. Further, it is important to realise that there are stages towards acceptance; for instance, individuals may progress from initial realisation to acknowledgement and onwards to a final acceptance [33]. Additionally, there is a need to understand in what ways defiance is undertaken by the patient. For example, to defy some patients may mean retaining their independence and engaging in activities that they did before illness; alternatively defiance may be expressed as hope in a cure. Thus, rather than categorising patients into particular stages or phases of adjustment [33] it is important that health care professionals consider what a patient can accept and in what ways they want or will defy the illness and this primarily relates to previous identities and meaningful activities.

Further to this, it is possible to identify the fact that the levels, or order, of hopes in a patient's life have several important outcomes. These include that (a) acceptance cannot be a generic term used in order to understand adjustment; rather it should be considered on an individual level, acknowledging the multidimensional nature of it as a concept. (b) Being able to accept one's prognosis does not mean that a patient has to accept a loss of their identity; rather identity can be maintained through illness, for example [26], from the past to the future and health care professionals need to beware of this if they are removing or challenging a patient's hope. (c) A patient can accept what is happening and at the same time defy an illness by remaining or retaining the important aspects of their identity.

### 4.2. Relief from Suffering

The current results identified the fact that individuals were often shocked and overwhelmed by what had happened; this feeling is one of three feelings that relates to hopelessness [31]; (1) the sense of the impossible that no matter what is tried their situation cannot be overcome; (2) the sense of “too-muchness” that an individual feels overwhelmed by what has happened and is unable to handle the situation; and (3) finally and most importantly an individual feels a sense of futility. Futility is identified as the heart of hopelessness for individuals [31] and it exists when there is an “*overwhelming improbability in the face of possibility*” (page 42) [45].

### 4.3. Implications for Health Care Professionals

Following this review of literature, several suggestions can be given to health care professionals that may help their patients. First, health care professionals may consider what aspects of identity and what skills or previous talents an individual had that can be used in future activities. In other words, health care professionals need to consider who the patient was before diagnosis/onset and consider the importance stroke survivors attach to returning to the activities, relationships and identities associated with their past self. This may be achieved through examining what a patient can accept or need to defy about their situation. Even having the possibility of hope in the future is important as it may also allow hope in the future and thus bring less suffering to the present [31]. Second, health care professionals must make sure that being realistic about a patient's prognosis and expectations does not prevent them from engaging in life and it may be that we can assist how they view and regard rehabilitation efforts to promote their ability to cope. For instance, using a wheelchair may be associated with hopelessness as it may imply that there is no hope for change; however, if a patient fully understands the purpose of using a wheelchair, which is to enable independence, which in turn can allow accessing rehabilitation, they can see that the best attempt to promote change may lie with this option. Thus, hope can be established through the ability to cope rather than the ability to be restored instantly. Third, health care professionals need to consider that the patient may not have had time to, or not be able to, reappraise their life situation [14] in a positive way or may have been unable yet to begin a search for new meaning and value in life [4], which can be a central and important aspect that relates to hope. This is important, because hope is generated by a positive reappraisal of one's situation; it is generated from seeing possibility in one's situation and it is generated by gaining a sense of purpose and establishing a routine. The most effective illustration of reappraising one's situation is perhaps found in observing how like-minded others with similar conditions have continued. Further to this, it may be that using such individuals for social support will provide access to a more positive adjustment to one's situation and identity. Finally, health care professionals are able to acknowledge that patients who have been diagnosed with a chronic condition may be vulnerable to hopelessness. In a deteriorating chronic neurological condition, hopelessness will be generated from a loss of the individual's high order hopes, and as the deterioration continues finally the condition will affect their low order hopes. Patients are often able to observe this before it happens; thus their future may become hopeless, or they may be able to see that there is a point in the future beyond which it will be recognised as hopeless. Health care professionals are required to understand this impact on the patient. However, health care professionals may use shorter term goals or aspects in the individual's life to consider as one strategy to overcome; alternatively they may consider the other aspects of life that can offer hope, that is, within more superficial hopes or the hope generated through having a sense of purpose in helping others, for instance, welcoming individuals who are newly diagnosed to a professional support group like the MS society [29].

## 5. Conclusion

A more generalised view of hope is called for in rehabilitation, beyond a pure focus on goals, moving towards understanding how the patients view themselves. A first step to help the patient is hearing how they consider the paradox of chronic illness, that is, what do they accept or need to defy? Further, once this is expressed, health care professionals need to consider in what ways it is possible to help individuals retain who they are and how they see themselves. Finally, health care professionals can aid this process by helping patients understand the importance of aspects of rehabilitation that promote coping rather than curing, as this will enable the individual to continue in their lives, helping continuity and ultimately allowing and promoting a sense of hope in the patient's present circumstances.

## Supplementary Material

Identifying the thematic structure of generalised hopes for individuals who have suffered a stroke.

## Figures and Tables

**Table 1:** Demographic information of individuals included.

Author	n	Gender, marital status, and age	Type of injury	COREQ summary (/32)
Arnaert (2006) [8]	8	4 male, 4 female 4 married, 1 widowed single, 1 divorced Age: 19–90 years (mean 60.1)	7 ischemic 1 hemorrhagic Stroke severity ranged from 3.0 to 11.5 on the Canadian Neurological Scale (CNS)	19/32

Bays (2001) [37]	9	3 male 6 female 6 married, 3 widowed Age: mean 68.2	6 right hemispheric strokes 2 left hemispheric strokes	20/32

Wiles (2002) [38]	13	8 male 5 female Marital status not mentioned Age: 41–79 years (mean 66)	No diagnostic information available.	21/32

Barker (2005) [36]	19	12 male 7 female 9 married Age: 42–82 years (mean 63.7)	12 left sided weaknesses 7 right sided weaknesses	21/32

Tutton (2012) [39]	10	7 male, 3 female No martial detail provided Age 37–72 years (median 63)	No diagnostic information available	19/32

Lutz (2011) [11]	19	11 male, 8 female No martial details provided Age: 33–84 years (mean 64)	No diagnostic information available	19/32

Kouwenhoven (2011) [40]	9	3 male, 6 female 5 married, 3 widow, 1 divorced Age: 30–75 years (mean 62.7)	3 left infarctions 5 right infarctions 1 right haemorrhage	22/32

Hartigan (2011) [7]	10	5 female, 5 male 3 married/with spouse 7 single Age: 70–83 years (mean 77)	No diagnostic information available	18/32

Cross (2010) [41]	10	10 female 4 married 2 nuns 4 widowed Age: 71–100 years (mean 79.8)	No diagnostic information available	22/32

Bright (2013) [10]	5	2 female, 3 male No martial details provided Age: 41–62 years (mean 50)	4 left infarctions 1 left haemorrhage	21/32

Note: studies denoted by the first author.

**Table 2:** The summary of correctly scored domains of the COREQ (Tong et al., 2007) [34] appraisal for the 4 included studies.

Author/year of publication	Domain 1 (/8) research team and reflexivity	Domain 2 (/15) study design	Domain 3 (9) analysis and findings	Total (/32)
Arnaert (2006) [8]	5	8	6	19
Bays (2001) [37]	4	8	8	20
Wiles (2002) [38]	4	12	6	22
Barker (2005) [36]	4	10	7	21
Tutton (2012) [39]	5	8	6	19
Lutz et al. (2011) [11]	4	9	6	19
Kouwenhoven (2011) [40]	4	11	7	22
Hartigan (2011) [7]	4	8	6	18
Cross (2010) [41]	4	12	6	22
Bright (2013) [10]	4	11	6	21

Mean	4.2	9.7	6.4	**20.3**
Median	4	9.5	6	**20.5**

Note: studies denoted by the first author.
