# Supplementary material for: Identifying a Framework for Hope in Order to Establish the Importance of Generalised Hopes for Individuals Who Have Suffered a Stroke
Source: Adv Med. 2014 Jun 29;2014:471874. doi: 10.1155/2014/471874 (PMC4590961; doi:10.1155/2014/471874)
Supplement: Supplementary file 1 — Identifying the thematic structure of generalised hopes for individuals who have suffered a stroke. [file 471874.f1.pdf]

Table 3 identifying the thematic structure of generalised hopes for individuals who have suffered a stroke

Theme	Sub-theme	Unit
Consideration of a patient's identit/ies	Loss of identity	"The changes in light of their sense of self and worried that in some ways this may be damaged or lost:<br>The other thing was this worry, am I still me. Afterwards, suddenly I have a lopsided face, everyone I have met so far have said I am still me (P8)." (5)<br>"Hopes around the body were often related to loss or reflected the change in their lives." (5)<br>"The massive effect on all aspects of their life" (5)<br>"Ingrid felt that she was not an efficient housewife anymore. Before, she could do housework for hours, now her abilities were limited and family members helped her doing the heavy tasks in the house. She described herself as an old lady" (7)<br>"For this group, life totally changed after stroke. They felt like they were victims and had lost themselves to the stroke…The participants described life as boring and empty without pleasure. The stories were described without colours, just greyness." (7)<br>"Hedda and Erik…had significant cognitive and physical limitations. The were middle aged, and had families with children living at home…A lot was at stake in terms of work, economical, situational and families" (7)<br>"Peter did no trust himself anymore. He was afraid that he would not be able to take full responsibility for himself….Peter felt vulnerable when a lot of people were present" (7)<br>"The experiences of three of the participants reflected this pathway; Peter, Ellen and Anne. For this group, life totally changed after stroke. They felt like they were victims and had lost themselves to the stroke; they were trapped in a different life" (7)<br>"You know, it's very humiliating to have to call somebody so you can go to the damn bathroom in the middle of the night. Just to go to the bathroom. Yeah, that's when you know you're really screwed up, when you have to call somebody to help you out with something that simple, something you take for granted your whole life. [P-13]" (6)<br>"I feel like I'm different from everybody else……I'm kind of not so happy about the person I've become. The one I am no. I sort of feel that I …felt like…kind of a geek. With a poor balance and stuff" (7)<br>"Erik felt degraded as an employee and man" (7)<br>"Participants…expressed enormous losses associated with arm impairment" (4)<br>"when the leg comes back [regains function] the only thing you learn to do is walk. But the number of thing you can do with an arm [are a lot greater]" (4)<br>"participants experience a significant amount of disruption to their identity" (10)<br>"a co-existing loss of identity influenced their experience of hope" (10)
	Stroke related changes that could redefining the patient's current identity	"I was a right handed man" (4)<br>"she was immediately assigned to a wheelchair" (4)<br>"The swiftness in which people became dependent on others for bodily support such as toileting, invasive devices such as naso-gastric tubes and catheters was often disturbing for participants. Stepping back from being independent and supporting others to accepting help was also a challenge" (5)<br>"stroke survivors began to acknowledge post-stroke impairments during this phase" (6)<br>"Once home, stroke survivors realised they had to depend on others for things they took for granted their whole lives" (6)<br>"The physical and cognitive changes after stroke improved early. Although there still were some limitations, they managed to live with them" (7)<br>"Nora experienced great difficulties understanding the content of texts as quickly as she did before" (7)<br>"After her stroke, Sigrid experienced being labile. She used to be a calm, balanced old woman. After stroke she had episodes where she all of a sudden turned angry, yelled at her husband or children, and then started crying." (7)<br>"Before the stroke she often went shopping and visiting cafe´s with her girlfriends. Now she only left home when it was crucial, and only with help from others." (7)<br>"After stroke Peter regained some of his walking capacity and at 6 months he was able to walk short distances using ski poles. At the 12-month interview his lung disease worsened and limited his ability to walk. Six months later his condition was even worse, and he was no longer able to do everyday chores he previously considered simple and obvious." (7)<br>"Having a stroke changed the way the participant felt about themselves. They became self-critical. Being dependent on others was difficult. They were old, living alone, and received restricted community support in relation to solving everyday issues" (7)<br>"By highlighting the past and comparing with the present, Peter expressed the experience of being less attractive, being of less use and therefore less valued." (7)<br>"My friends who see me often, they say that the can see that I've lost the spirit that I had for thinking positively…It isn't me…it's hurting my marriage, its hurting most things" (7)<br>"Erik experienced cognitive changes in relation to problem solving concentrating, and strategically thinking…on the one hand, he believe he would be the person he was before…on the other he realised he was a different man" (7)<br>"Falls generally occurred when caregivers were helping the patients transfer to and from the

		bed or wheelchair or when the patients lost their balance or tried to transfer without assistance.” (6)<br>“Getting back to a normal life was also often qualified by the context of the uncertainty of life from now on” (5)
	Restoring their past identity or obtaining a valued identity - generic concept that links identities and returning to valued or pre-stroke lives	“they soon regained balance and found a restored self.” (7)<br>“Jon was a truck driver, for whom getting his driving license back was the central goal. For his<br>family and friends these seemed unattainable, but 12 months after the stroke he had regained his driving licence for regular cars…At 18 months he was in a customised programme, and had started to work as a ‘‘henchman’’ on the truck he used to drive” (7)<br>“at 12 months, Nora started to work full time” (7)<br>“The experience of finding a restored self” (7)<br>“At 18 months Nora said: ‘‘I don’t think I can be better than this! I feel like I am the one I was.’’(7)<br>“In every interview, peter talked about life before stroke and things he had achieved.” (7)<br>“At the time the participants were focusing on the goal of regaining self” (7)<br>“To Erik…the role as a leader in a big company was a large part of his identity” (7)<br>“Hedda and Erik fought hard to regain self and to become as they were before the stroke.” (7)<br>“All participants expressed a future goal or desire…which collectively was the desire to return to normal pre-stroke life” (1)<br>“after 12 months they started to question their expectations [to be restored to pre-stroke]” (7)<br>“their definitions of recovery were often intensely personal…[and included]…a familiar identity” (4)<br>“A good recovery was to … to get your life again” (4)<br>“She stopped smoking, started on a healthy diet, and began to exercise. To her, having a stroke became an important ‘‘wake up call.’’” (7)<br>“Having hope appeared to provide stability that allowed participants to maintain hope that improvement was possible while their waited for change or a return to “normal” (10)<br>“Broad hopes arose from what was meaningful to the individual and their sense of self. The focused on roles in life, sense of identity” (10)<br>“at times hopes and expectations for recovery dominated participants views” (10)<br>“Matthew…didn’t want to be the person he was before the stroke and thus, at one level, welcomed the chance to do things differently” (10)
	Return to normal or pre-stroke lives - generic concept that links identities and meaningful activities	“All participants expressed a future goal or desire…which collectively was the desire to return to normal prestrike life. This recurrent idea was indicated though statement about the importance of work, school and leisure activities” (1)<br>“The youngest participant (S3) identified a desire to return to their prestrike life “To go back to school” (1)<br>“Another participant (S3) described the importance of work, “well I hope to go back to work. I really love my job.” (1)<br>“I [hoped I] would be able to move and feel like I used to…that I would be back like I was before I had the stroke” (2)<br>“the notion of recovery was understood by patients as being a return to ‘normal’ life which meant a resumption of the activities that they undertook before they had the stroke” (3)<br>“the expectation that full recovery could be achieved emerged at all three interviews” (3)<br>“A good recovery was to have some return of movement and feeling, use of the hand, to do what you want to do” (4)<br>“Hope for recovery reflected the determination to move forwards and get back to normal or to meet aspirational goals.” (5)<br>“Some participants hoped to get back to how things were before” (5)<br>“The drive to get back to normal was strong for some” (5)<br>“The implied promise of inpatient rehabilitation was that patients would not be discharged home<br>until they were able to return to their pre-stroke lives.” (6)<br>“Stroke survivors’ views of “getting better” meant returning to their pre-stroke activities and life: “I expect that I’ll go back to doing everything I did before this all happened” [P-6].” (6)<br>“Regaining the driving licence was an important part of achieving independency” (7)<br>“To get back to where I was last week [P5]” (8)

		"To be able to use my (right) hand [P7]" (8)<br>"To be able to do normal things [P8]" (8)<br>"Despite this, several participants had a positive outlook on their recovery post stroke" (8)<br>"The participants spoke about the importance of returning to the 'normal' life they had before their stroke…Normal life was related to regaining control in three areas; personal care, everyday responsibilities and social functioning." (8)<br>"Two participants (S5-S6) expressed the desire to go home as part of their vision of hope… "Home is where I am normal" (1)
	Religious or spiritual dimension of identity	"the spiritual dimension of hope was identified by 2 participants" (1)<br>"well certainly people with complete faith have something to fall back on when they need it" (1)<br>"all participants were members of Christian churches" (2)<br>"…a strong faith in God…when God closes one door He opens another" (2)<br>"I guess my faith is at the bottom of everything. I have always been a Christian since I was a child. I have never waivered in my faith.…You know like they say you have to have blind faith and that's what I have blind faith. I know that you can't prove everything. But I don't expect it to be proven. But the blind faith has really kept me going." (9)<br>"Broad hopes arose from what was meaningful to the individual … They focused on …faith" (10)
Meaningful activities, experiences and interactions	Meaningful interactions	"anything anyone who gives you strength or support when you need it and that's hope" (1)<br>"the wife and I and the other couple…singing and playing together" (2)<br>"my friends have always been present" (2)<br>"I have tended to put faith in God and other people" (2)<br>"these [meaningful] activities identified [as]… playing with children" (3)<br>"[recovery was linked to interactions with participants] spouse, family, friends, and community, other stroke survivors and health professionals" (4)<br>"a number of participants were unable to imagine how recovery was possible for those who did not have a spouse" (4)<br>"stroke survivors drew on support from others" (4)<br>"Support and understanding from the surroundings were crucial to all of the participants." (7)<br>"Anne experiencing that close acquaintances stopped contacting her after the stroke" (7)<br>"Anne had problems with communication, especially finding words and expressing her thoughts. She avoided social gathering and situations where she had to talk to people who were not close to her" (7)<br>"Challenges in relation to close relationships and intimacy were only described by one participant..Hedda felt trapped by her husband's expectations….after the stroke she felt that he let her down. His expectations, particular in terms of sexual activity, increase and tings became more difficult" (7)<br>"Medical support systems, rehabilitation programs, and other stroke survivors were all described as playing an integral role in influencing hope" (9)<br>"Support systems include medical staff, rehabilitation therapists, family, and friends. They are an intricate network that work together to motivate, inspire, guide, and care for the stroke survivors [e.g.,]…"I think knowing I have such support. Just knowing that if I am having a problem all I have to do is pick up the phone, so you're not alone."…"[My granddaughter and friend] give me hope."…"[My husband] is always there for me." " (9)<br>"hope was gained from multiple sources, with "other people" being the most common source of hope, although they could also pose a risk to hope" (10)<br>"The process of talking about hope helped Adrienne "realise there was hope there". Rehabilitation staff provided hope for some people" (10)

	Returning to meaningful activities - generic concept that links identities and returning to valued or pre-stroke lives	"well to be able to do things for myself again and return to my activities...going back to my home and perhaps playing bridge" (S6)" (1)<br>"that I could, get around, you know…regaining mobility for th leg and arm…playing golf or playing my mandolin again…drive a care" (2)<br>"being with my family and friends [is essential]" (2)<br>"these [meaningful] activities identified [as]…cycling, playing golf, walking" (3)<br>"their definitions of recovery were often intensely personal…[and included]…valued activities and lifestyle choices" (4)<br>"I just want to hold a hammer in it…you have to hands…woodwork in the shed" (4)<br>"Many participants wanted to move on from sitting and waiting and get back to walking and doing things for Themselves" (5)<br>"one lady wished to go skiing with the family" (5)<br>"Another said, "I just wanted them to bring me in here [IRF] and teach me to walk, so I could walk out of here" [P-1]. This survivor then expressed that even if this level of recovery took some time to occur, he would "eventually…be able to walk alone, by myself, within a year." (6)<br>"The experience of being free distinct from being dependent on others was crucial to the participants." (7)<br>"To Erik, the main goal was to return to work" (7)<br>"To be able to check the cows…To work and care for collie dogs [P2]" (8)<br>"use the computer again…write computer programmes [P3]" (8)<br>"To be able to walk and talk again [P3]" (8)<br>"To recover 100% of my speech…to get home…to do some gardening [P4]"<br>"To be able to get up and get dressed myself" [P6] (8)<br>"To travel with my wife…To play golf [P7]" (8)<br>"To drive and play golf[P8]" (8)<br>"to be able to walk and talk and get home [P10]" (8)<br>"Participants looked forward to being able to return to their pre-stroke responsibilities; "I'm not fully sure till I get home…I am feeling good right now and want to do those things (jobs on the farm) I'll know more when I'm there and see can I do the jobs" [P2]" (8)<br>"hopes of achieving a level of recovery and function that would allow them to return home Independently" (6)<br>"To be independent [P1]" (8)<br>"Participants yearned to remain optimistic for the future and motivated to accomplish normal activities of everyday life" (8)<br>"Individuals up to 4 years post stroke remained hopeful for future improvement in both specific and general areas." (9)<br>"more specific hopes such as continuing theological study or passing values onto their children" (10)<br>"There was a high degree of dependency on others for all aspects of care and patients really struggled to make their bodies work: 'he tried to wash his face but his arm started shaking and he just held the cloth<br>on his face' (FNp3)" (5)<br>"The nature of hope was presented in the context of having a stroke and the impact on the patient's body and ability to function." (5)<br>"participants experience a significant amount of disruption to…engagement in meaningful activities" (10)

	Revaluating and redefining meaningful activities	"Participant S2 had no permanent sequelae and was able to perceive her stroke as a positive life transforming event. The stroke caused her to shirt her focus from present goals and objectives to a more global view of the future for herself and her son" (1)<br>"…I am a little selfish when I see some of my friends who are still in a wheelchair…Ii realise I have more than a lot of people have" (2)<br>"I cant do the things I used to but I can do new things" (2)<br>"Some participants hoped to get back to… find new ways of living or being." (5)<br>"In the context of uncertainty, participants identified the importance of living for the moment and making the most of life as it is in the present. For them being alive was meaningful for them and they had a strong drive to make the most of life." (5)<br>"Both staff and participants used goals as a way of being hopeful about the future" (5)<br>"They often reframed longer term hopes by focussing on immediate goals. However, the staff were constantly evaluating and reframing the future for patients. If it became apparent that walking was not possible, there was a refocusing of hopes on home and what might be possible" (5)<br>"They became aware of what they described as the important things in life: being grateful for the things they have achieved, taking care of own health, and being together with their loved ones." (7)<br>"The women expressed the importance of taking it day by day and being happy with any progress they were making. [e.g.]…"No, I just went day to day and did what I was told."…"I just take every day as it comes and I make the best of every day."" (9)<br>"by accepting the limitations and finding new solutions they were no longer trapped, but had the ability to do more or less the same things they did before the stroke." (7)<br>"Broad hopes arose from what was meaningful to the individual … They focused on …outlook on life" (10)
	Meaningful Religious practices	"a sense of relatedness and connected ness with others, nature and with God" (S2)" (1)<br>"feeling that God is behind you all the way" (2)<br>"I believe there is a God in heaven and an all-powerful God who all things are possible through Him" (2)<br>"Approximately half of the participants stated that spirituality was not a major influence on their hope and recovery, whereas the other half felt it had played a prominent role….[e.g.]…"I just know that with the belief in God that he will make things better has made my life a lot easier."… "You pray for things and hope that the Lord will hear you and of yourself. We can do nothing but with prayer and hope, it all helps along."" (9)
	Superficial hopes related to an individual's daily routine and meaning	"all but one patient received out-patient physiotherapy one or twice a week" (3)<br>"navigating rehabilitation services" (4)<br>"participants also looked to health care professionals for support" (4)<br>"exercise was seen as a means to physical recovery" (4)<br>"participants agreed that attendance at therapy,…helped maintain the motivation to keep going" (4)<br>"the option of inpatient (acute) rehabilitation was presented to patients and family members" (6)<br>"The participants emphasised the importance of being able to participate, contribute, and do something useful in their daily life." (7)
The experience of suffering and need for relief	Experiences of acute suffering	"all had suffered a sudden onset with neurological deficit and had their lives incomprehensibly turned upside down" (1)<br>"Vulnerability and horror were expressed as people watched their bodies not working as they should" (5) "the emotional impact was expressed in a verbal and nonverbal way, including a variety of overwhelming feelings, such as fear, anxiety, anger, surprise, and hopelessness" (1)<br>"one young male participant (S1) 19 years of age said, "it was terrifying, very terrifying. I knew something terrible was happening" (1)<br>"Another participant (S8) said "Overwhelmed, I'm not even sure I was frightened just overwhelmed…once I realised what was wrong, then I would not speak. It was fear of what was happening" (1)<br>"The constant reminder of the losses and the frustration of repeated failure overwhelmed those with severe paresis" (4)<br>"Simple activities, like sitting out in a chair, caused discomfort: 'she says she has a pain that cuts her in half like a knife'" (5)<br>"Of great significance for many was the loss of the ability to swallow a cup of tea or the ability to walk." (5)<br>"They experienced stress, confusion, fear, and loss of control and began to wonder what would happen next…With the crisis of stroke, the focus was on survival and determining what steps should be taken next" (6)<br>"Initially they expressed feelings of shock and fear related to loss of bodily control" (8)<br>"when uncertainty dominated, the future became restricted and the focus returned to the present and, focusing on "just getting through" (10)
	Considering the impact of the	"One young male participant (S1), 19 years of age, said I saw my whole life in front of my eyes, it was awful" (1)

	stroke on accessibility of an individual's future and identity	"I realise that in the future when I'm feeling tired maybe I should not push myself as I do now.. maybe I'll take things a little more easily and ill relax when I'm tired" (S1) " (1)<br>"Participants' hopes were therefore experienced within the impact of the stroke on their body and their lives" (5)<br>"Getting back to a normal life was also often qualified by the context of the uncertainty of life from now on" (5)<br>"The majority of patients believed that they would truly know the extent of their stroke once they returned home. "I would love to be at home to try it out, I really feel like you are under observation here, you would love to do things for yourself like, see how I go…It is at home you would really have to test it……" (P1)." (8)<br>"in the acute phase of their stroke they appeared to be more caught up in their present reality and its associated fears , frustrations, and anxieties, and therefore unable to foresee the future" (1)<br>"If I am too incapacitated and I cannot go home then I would go to a nursing home. I've thought about that over the last few days" (1)<br>"some patients who were resistant to accepting that a return to pre-stroke activity was unlikely" (3)<br>"The participant observation noted that for many there seemed to be no hope of recovery: 'for Jane, Ellie and Ann there is really little chance of great improvement so little chance of a future for them' (FN2 p 4)." (5)<br>"it was noted that financial constraints within the service also had a role to play in determining the amount of out-patient therapy received" (3)<br>"For patients who lived alone and were not able to identify a primary family caregiver who could provide care post discharge, this was also a time to begin thinking about and planning for their future options" (6)<br>"They believed that when they got home, things would return to normal and life could resume its previous routine, with few or minor changes. However, at some point during the rehabilitation process, family caregivers began to realize the stroke survivors were going to be discharged even if they had not met their functional goals and that they (the caregivers) needed to prepare for the stroke survivors' post discharge limitations and resulting needs" (6)<br>"Patients, themselves, were also alarmed if they felt they were dismissed prematurely. Being home also meant a drastic reduction in therapy, from 3 hours per day to a few hours 1 or 2 days a week." (6)<br>"Options for stroke survivors who lived alone were limited if they did not have the support of<br>family and/or friends for post discharge care." (6)<br>"All participants experience uncertainty about what their future might be..[at times] uncertainty would dominate [participants views]" (10)

(1) Arneart et al (2006), (2) Bays (2001), (3) Wiles et al (2002), (4) Barker & Brauer (2005), (5) Tutton et al (2011), (6) Lutz et al (2011), (7) Kouwenhoven, et al (2011), (8) Hartigan et al (2011), (9) Cross and Schnieder (2010), (10) Bright et al (2013).
